# New Taxa of Filamentous Cyanobacteria from Freshwater Habitats in China: Description of *Neoleptolyngbya* gen. nov. with Two Species, and *Pseudoleptolyngbya wuhanensis* sp. nov

**DOI:** 10.3390/microorganisms14010072

**Published:** 2025-12-29

**Authors:** Fangfang Cai, Jiaxin Chen, Shuheng Li, Xiaochuang Li, Renhui Li

**Affiliations:** 1 Hubei Key Laboratory of Animal Nutrition and Feed Science, Wuhan Polytechnic University, Wuhan 430023, China; 2 College of Resources and Environment, Anqing Normal University, Anqing 246011, China; 3 School of Life and Environmental Sciences, Wenzhou University, Wenzhou 325035, China

**Keywords:** cyanobacteria, *Neoleptolyngbya*, *Pseudoleptolyngbya wuhanensis*, Leptolyngbyales, new taxa, polyphasic methods, biodiversity

## Abstract

Three cyanobacterial strains (CB-4, GQSK-2, and LHH-2) with thin, simple filaments were isolated from freshwater habitats in China. In this study, a polyphasic approach, integrating 16S rRNA gene phylogenetic analyses, p-distance calculation, 16S-23S ITS secondary structures, and morphological and ecological observations, was employed to resolve the taxonomic status of these strains. The results confirmed the existence of two new species within a novel genus (*Neoleptolyngbya*) and one additional new species of *Pseudoleptolyngbya* (Leptolyngbyaceae): *Neoleptolyngbya gaoqiuensis* sp. nov. (strain GQSK-2), *Neoleptolyngbya chanbaensis* sp. nov. (strain CB-4), and *Pseudoleptolyngbya wuhanensis* sp. nov. (strain LHH-2). In the 16S rRNA phylogeny, strains GQSK-2 and CB-4 formed a well-supported, independent lineage sister to the *Leptolyngbyopsis* clade, while strain LHH-2 clustered with two recognized *Pseudoleptolyngbya* species in a distinct clade. Sequence similarity analyses revealed that the 16S rRNA gene sequences of GQSK-2 and CB-4 shared a maximum similarity of 94.2% with those of phylogenetically related established genera, and the 16S rRNA gene sequence of LHH-2 exhibited a maximum similarity of 95.7% with its closest *Pseudoleptolyngbya* relatives, both values below the threshold for cyanobacterial species/genus delineation. Furthermore, comparative analysis of the 16S–23S ITS secondary structures between the three strains and their respective reference strains revealed significant species-specific differences, providing additional evidence for their taxonomic novelty. The discovery of these novel taxa enriches the cyanobacterial diversity in China and lays a theoretical foundation for the conservation and sustainable utilization of algae resources.

## 1. Introduction

As one of the oldest prokaryotic lineages on Earth, cyanobacteria play crucial ecological roles in freshwater, marine, brackish, and terrestrial environments. They contribute to oxygenic photosynthesis, global carbon/nitrogen cycling, and the production of bioactive secondary metabolites [1,2,3]. Despite their ubiquitous presence across diverse environments, a large portion of cyanobacterial biodiversity remains poorly characterized [4,5]. Thus, comprehensive research and accurate taxonomic identification of cyanobacteria are of great significance [6,7]. The adoption of a polyphasic approach has greatly advanced cyanobacterial taxonomy, effectively addressing the limitations of traditional morphological classification [8,9]. This polyphasic approach establishes a framework for resolving the challenges of identifying cryptic species; many cyanobacteria that are morphologically identical exhibit significant genetic divergence [10,11,12]. This is particularly true for thin filamentous cyanobacteria (with cell diameter < 3.5 μm). In the early classical classification system, all filamentous cyanobacteria lacking heterocytes or akinetes were categorized under the order Oscillatoriales; within this taxon, thin filamentous cyanobacteria were placed in the family Pseudanabaenaceae and the genus *Leptolyngbya* Anagnostidis and Komárek [13]. However, the application of cell ultrastructure and 16S rRNA molecular sequence analysis to cyanobacterial classification revealed a more complex evolutionary relationship among these filamentous taxa [14]. For example, thylakoids in *Oscillatoria*-like groups are often radially arranged, whereas thin filamentous cyanobacteria typically have parietal thylakoids, a feature shared with the order Synechococcales [15]. In Hoffmann’s proposed system, the family Pseudanabaenaceae was elevated to the order Pseudanabaenales, which, together with Synechococcales, was classified under the subclass Synechococcophycideae [15]. In the subsequent eight-order classification system, Pseudanabaenales was subsumed under Synechococcales; this order included all small unicellular coccoid and thin filamentous cyanobacteria with simple morphologies, and thin filamentous cyanobacteria with sheaths were assigned to the family Leptolyngbyaceae [16]. Mai et al. conducted a taxonomic revision of the family Leptolyngbyaceae; the findings demonstrated that taxa previously classified under Leptolyngbyaceae can be further delineated into four distinct family-level clades, that is, Leptolyngbyaceae, Oculatellaceae, Prochlorotrichaceae, and Trichocoleaceae [17]. A groundbreaking revision of cyanobacterial taxonomy in 2023 proposed a 20-order system, in which the four previously recognized family-level lineages of thin filamentous cyanobacteria were elevated to the order rank: Leptolyngbyales, Oculatellales, Nodosilineales, and Prochlorotrichales [18].

According to the revised cyanobacterial order and family classification by Strunecký et al., the newly established order Leptolyngbyales Strunecký and Mareš comprises thin filamentous cyanobacteria (<3 μm in width) with simple morphology and facultative sheaths [18]. This order includes families such as Leptolyngbyaceae (containing genera *Leptolyngbya* and *Phormidesmis* Turicchia, Ventura, Komárková & Komárek), Trichocoleusaceae (containing only the genus *Trichocoleus* Anagnostidis), and Neosynechococcaceae (containing the single described genus *Neosynechococcus* Dvořák, Hindák, Hasler & Hindáková) [18]. Compared with other described cyanobacterial orders, members of Leptolyngbyales are more frequently found in soil and other non-aquatic/terrestrial environments [18]. Filaments of Leptolyngbyaceae typically range from 1.5 to 2.5 μm in width (with a maximum of up to 4.5 μm). Genera such as *Leptolyngbya*, *Phormidesmis*, *Leptodesmis* Raabová, Kovacik & Strunecký, *Myxacorys* Pietrasiak & Johansen, *Alkalinema* Vaz et al., and *Chamaethrix* Dvořák et al. have cylindrical cells that become isodiametric or wider than long after division [19,20,21,22], while *Stenomitos* Miscoe & Johansen, *Onodrimia* Jahodárová, Dvorák & Hašler, and *Scytolyngbya* Song & Li have cells that remain longer than wide [23,24,25].

The newly established genus *Pseudoleptolyngbya* Hentschke gen. nov. was isolated from freshwater environments in Figueira da Foz, Monchique, and Coimbra, Portugal [26]. This genus is characterized by homocytous, thin, cylindrical trichomes with distinct constrictions at cross-walls, enveloped by delicate colorless sheaths. Morphologically and ecologically indistinguishable from *Leptolyngbya*, *Pseudoleptolyngbya* can only be reliably differentiated through molecular characterization. Currently, this genus comprises two described species: *Pseudoleptolyngbya figueirensis* (type species) and *Pseudoleptolyngbya monchiquensis*.

To further explore the diversity of thin filamentous cyanobacteria, this study used a polyphasic approach—incorporating morphological observation, ecological characterization, 16S rRNA gene phylogenetic analysis, and 16S–23S rRNA intergenic spacer (ITS) region analysis—to identify cyanobacterial isolates from subtropical and temperate regions of China. Three cyanobacterial strains, namely, CB-4, GQSK-2, and LHH-2, were successfully purified and cultivated. Therefore, we herein describe the new taxa in the Leptolyngbyaceae: a new genus, *Neoleptolyngbya* gen. nov., which encompasses two new species: *Neoleptolyngbya gaoqiuensis* sp. nov. (GQSK-2) and *Neoleptolyngbya chanbaensis* sp. nov. (CB-4); a new species in *Pseudoleptolyngbya*, *Pseudoleptolyngbya wuhanensis* sp. nov. (LHH-2).

## 2. Materials and Methods

### 2.1. Sampling, Isolation, and Cultivation

Cyanobacterial strain CB-4 was collected in May 2023 from a shallow freshwater lake in Chanba National Wetland Park, Xi’an City, Shanxi Province, China (34°26′31.61″ N, 108°56′00.24″ E). The strain LHH-2 was isolated from shallow freshwater in Lotus Lake (31°90′60.59″ N, 118°6′60.54″ E) in Wuhan, Hubei province, China, in 2022. The strain GQSK-2 was obtained from plankton sample collected in Gaoqiu Reservoir, Nanyang city (33°0′3.8″ N, 112°31′45.4″ E), Henan province, China, in September 2023. The three studied strains were isolated from a plankton net sample. Unialgal cultures were obtained through serial washing using sterile Pasteur pipettes under 40× magnification (Olympus IX73, Tokyo, Japan). The purified filaments were maintained in BG11 liquid medium at 25 °C under cool-white fluorescent illumination (30 μmol photons m^−2^ s^−1^) with a 12:12 h light/dark photoperiod for several weeks. Subsequently, the pure filamentous strains were isolated and transferred to glass tubes containing 10 mL of BG-11 medium. Living cultures of these cyanobacteria are preserved at Wuhan Polytechnic University (Wuhan, China), and the dry material of these strains was stored in the Freshwater Algal Culture Collection of the Institute of Hydrobiology, Chinese Academy of Sciences (Wuhan, China).

### 2.2. Morphological and Ultrastructural Characterization

The morphological characteristics of these three isolated strains were observed using a Nikon Eclipse 80i microscope (Nikon, Tokyo, Japan). Microscopic imaging of cultured filaments was performed using a Nikon Eclipse microscope equipped with NIS-Elements imaging software (version 3.2D; Nikon Corporation, Tokyo, Japan). Morphologically related parameters were obtained by measuring the width and length of vegetative cells and filaments in over 100 individuals per strain.

For ultrastructural analysis, the strains CB-4 and GQSK-2 were initially fixed in 2.5% glutaraldehyde prepared in 0.1 M phosphate buffer (pH 7.2) at 4 °C for 3 days. Subsequently, they were rinsed with the same buffer, post-fixed in 1% osmium tetroxide for 2 h, and rinsed again. Dehydration was carried out using a graded ethanol series (20%, 50%, 70%, 90%, and 100%), followed by embedding in Spurr’s resin. Ultrathin sections were stained with 2% uranyl acetate and lead citrate prior to observation under a Hitachi HT-7700 transmission electron microscope (Hitachi, Tokyo, Japan) operating at 80 kV.

### 2.3. DNA Extraction, PCR Amplification, and Sequencing

DNA extraction of the strains was performed using a modified cetyltrimethylammonium bromide (CTAB) method [27]. Amplification of the 16S rRNA gene and the 16S–23S rRNA ITS region was achieved with primers PA [28] and B23S [29]. The PCR mixture included the following components: 25 μL of Premix Taq, 4 μL of template DNA, 1 μL of each primer, and 19 μL of ddH_2_O. Amplification was conducted under the following thermal profile: 95 °C for 3 min; 34 cycles of 94 °C for 30 s, 58 °C for 30 s, and 72 °C for 2 min; with a final 5 min at 72 °C. The resulting products were subsequently purified and recovered with an Omega kit (Omega, Norcross, GA, USA). The sequencing was conducted with an ABI 3730 automated sequencer system (PerkinElmer, Waltham, MA, USA), and the obtained sequences have been submitted to the NCBI GenBank under accession numbers PV911207, PV911208, PV911209, PV911210, PV911211, PV911212, PV892732, PV892733, and PV892734.

### 2.4. Phylogenetic Analyses

For phylogenetic placement, the obtained 16S rRNA gene sequences of the isolates were aligned with representative reference strains from Pseudanabaenales, Oculatellales, Nodosilineales, and Leptolyngbyales. Additional sequences were retrieved from the NCBI GenBank database using BLAST (NCBI, http://www.ncbi.nlm.nih.gov/genbank/, accessed on 10 November 2025) algorithms to ensure robust comparative phylogenetic analysis. A total of 233 retrieved 16S rRNA gene sequences (The 16S rRNA gene sequences of all strains used for phylogenetic analysis are listed in Table S1) were subjected to pairwise alignment using MAFFT v7.312 [30] under the auto-selected FFT-NS-I strategy, and the resulting alignment (1084 nucleotides) was subsequently inspected visually using MEGA v7.0.14 [31]. Phylogenetic trees were reconstructed using both Maximum Likelihood (ML) and Bayesian Inference (BI) methods. The ML analysis was conducted with IQ-TREE v1.6.12 [32], with robustness assessed by 1000 bootstrap replicates. The BI analysis was performed using MrBayes v3.2.6 via the CIPRES Science Gateway platform [33]. For Bayesian analysis, two independent runs were performed, each with four Markov chains, for 5 million generations, with a burn-in fraction of 25% and sampling every 1000 generations. The Bayesian MCMC analyses were run until the average standard deviation of split frequencies fell below 0.01, which was set as the convergence criterion and indicates sufficient sampling of the posterior distribution. The phylogenetic trees were rooted using the outgroup *Gloeobacter violaceus* PCC 7421 and viewed by FigTree v1.4.4 [34]. The 16S rRNA gene sequence similarity was calculated as 100 × (1 − *p*-distance) in MEGA v7.0.14 [31].

### 2.5. 16S–23S ITS Secondary Structure Analysis

Prediction of the ITS secondary structures (D1–D1′, Box–B, and V3 helices) for the studied and related strains was performed with m-Fold webserver [35] and subsequently redrawn using Adobe Photoshop CS6 Version 13.0.

## 3. Results

### 3.1. Molecular and Phylogenetic Analysis

**Figure 1:**
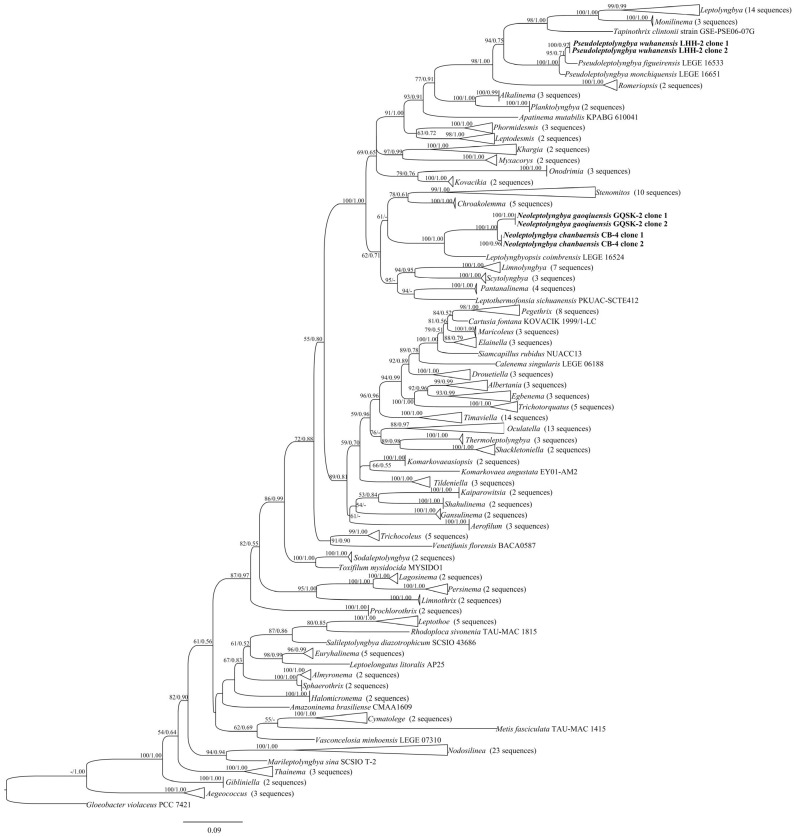
Maximum likelihood (ML) phylogeny of cyanobacterial 16S rRNA sequences, including the studied strains. Low-support nodes (ML < 50%; BI < 0.50) are collapsed. The novel filamentous strains characterized herein are shown in bold.

In the order Leptolyngbyales, all genera formed a monophyletic clade with strong support. Strains CB-4 and GQSK-2 clustered into a novel, well-supported clade (designated as *Neoleptolyngbya*), with a 100% ML bootstrap value and a 1.00 BI posterior probability (Figure 1). *Neoleptolyngbya* is closely related to *Leptolyngbyopsis*, a freshwater genus characterized by thin, cylindrical, constricted trichomes, and colorless and thin sheaths. The 16S rRNA gene sequence analysis showed that *Neoleptolyngbya* shares 93.5–94.2% similarity with *Leptolyngbyopsis* and <94% similarity with other closely related genera. Within the *Neoleptolyngbya* clade, the percentage similarity based on 16S rRNA genes between the strains CB-4 and GQSK-2 is 98.2% (Table 1).

The genus *Pseudoleptolyngbya* also formed a monophyletic clade. Two clone sequences of strain LHH-2 clustered with two *Pseudoleptolyngbya* species, with a 100% ML bootstrap value and a 1.00 BI posterior probability (Figure 1), and formed a distinct independent branch within the clade. The strain LHH-2 is closely related to *Pseudoleptolyngbya figueirensis*. The 16S rRNA gene sequence analysis revealed that LHH-2 shares 94.7% similarity with *Pseudoleptolyngbya monchiquensis* LEGE 16651 and 95.7% similarity with *Pseudoleptolyngbya figueirensis* LEGE 16533 (Table 1).

### 3.2. Taxonomic Descriptions

*Neoleptolyngbya* J.X. Chen & F.F. Cai gen. nov.

Diagnosis: Phylogenetically, *Neoleptolyngbya* is most closely related to *Leptolyngbyopsis* but can be morphologically distinguished from this sister genus by its wider cell width. Additionally, *Neoleptolyngbya* exhibits low 16S rRNA gene sequence similarity (Table 1) and distinct 16S–23S ITS secondary structures compared to other genera in *Leptolyngbyaceae*, supporting its recognition as a novel genus-level lineage.

Description: Thallus blue-green. Filaments solitary or in clusters, straight or curved, without false branching. Sheaths firm, transparent, colorless, tightly adherent to cells. Trichomes isodiametric, slightly constricted at cross-walls. Cells cylindrical, isodiametric, or slightly longer/shorter than wide. Parietal thylakoids. Apical cells elliptical to obtusely rounded. Gas vesicle absent. Reproducing via trichome fragmentation with or without necridia.

Etymology: The genus name “*Neoleptolyngbya*” refers to its morphological similarity to *Leptolyngbya*, despite their distant evolutionary relationship.

Type species: *Neoleptolyngbya gaoqiuensis*.

**Figure 2:**
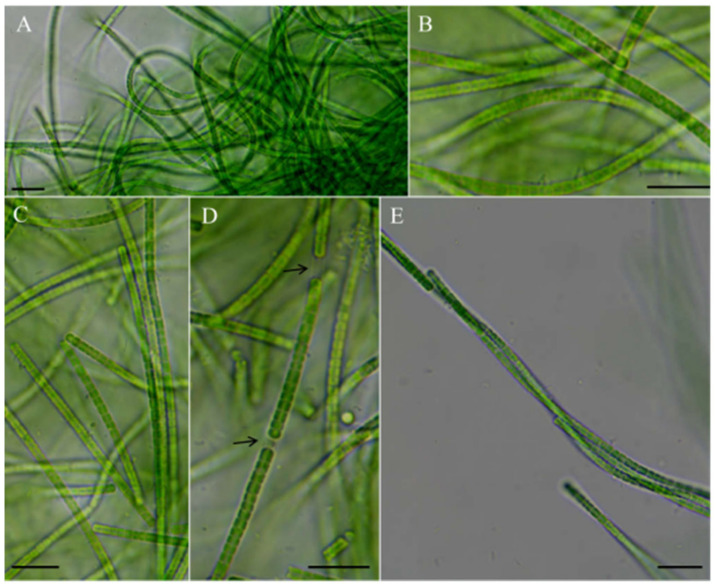
Micrographs of *Neoleptolyngbya gaoqiuensis* GQSK-2: (**A**) general view of microcolonies; (**B**,**C**,**E**) trichomes without sheath; (**D**) trichomes with sheath. Arrows indicate sheath. Scale bars = 10 μm.

*Neoleptolyngbya gaoqiuensis* J.X. Chen & F.F. Cai sp. nov. (Figure 2).

Diagnosis: Morphologically and phylogenetically, *Neoleptolyngbya gaoqiuensis* is the most similar to *Neoleptolyngbya chanbaensis* but differs in low 16S rRNA gene sequence similarity, as well as the composition and secondary structure of the ITS region.

**Figure 3:**
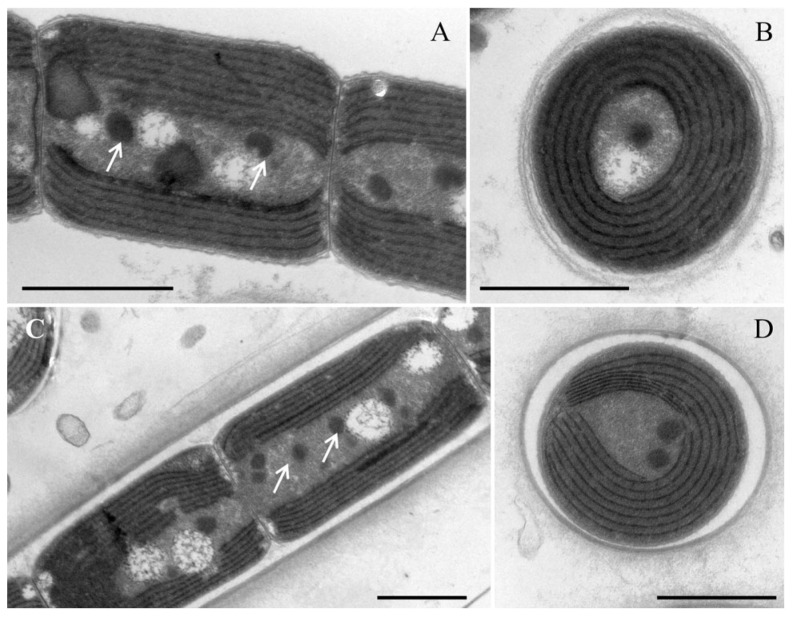
Transmission electron micrograph of the strain *Neoleptolyngbya gaoqiuensis* GQSK-2 and *Neoleptolyngbya chanbaensis* CB-4: (**A**) longitudinal section of a trichome in *Neoleptolyngbya gaoqiuensis* GQSK-2; (**B**) cross section of the cell in *Neoleptolyngbya gaoqiuensis* GQSK-2; (**C**) longitudinal section of a filament in *Neoleptolyngbya chanbaensis* CB-4; (**D**) cross section of the cell in *Neoleptolyngbya chanbaensis* CB-4. Arrows indicate granule. Scale bar = 1 μm.

Description: Thallus bright blue-green, floating or attached to the substrate. Filaments solitary or in clusters, straight, or curved. Sheaths often invisible, occasionally with firm, transparent, colorless sheaths. Trichomes with indistinct cross-wall constrictions and an isopolar organization. Cell cylindrical, longer or shorter than wide, 1.3–4.1 μm long, 2.0–3.6 μm wide. Parietal thylakoids (7–8 per cell, Figure 3A,B). Granules occasionally present in the cells (Figure 3A,B). Gas vesicle absent. Necridia present (Figure 2D). Apical cells elliptical to obtusely rounded. Reproduction by trichome fragmentation.

Holotype here designated: The dry specimen of strain GQSK-2 was deposited in the Freshwater Algal Herbarium (HBI), Institute of Hydrobiology, Chinese Academy of Science, Wuhan, China, as specimen No. GQSK202208.

Type locality: The strain GQSK-2 was collected in September 2023 by Chen Jiaxin from a water body near Gaoqiu Reservoir—a freshwater reservoir located in Nanyang City, Henan Province, China (coordinates: 33°0′3.8″ N, 112°31′45.4″ E). The strain exhibited a free-floating growth habit on the water surface.

Habitat: Freshwater.

Reference strain: GQSK-2 (Freshwater Algal Herbarium (HBI), Institute of Hydrobiology, Chinese Academy of Science).

Etymology: The name of new species “gaoqiuensis” refers to the locality where the strain was isolated.

**Figure 4:**
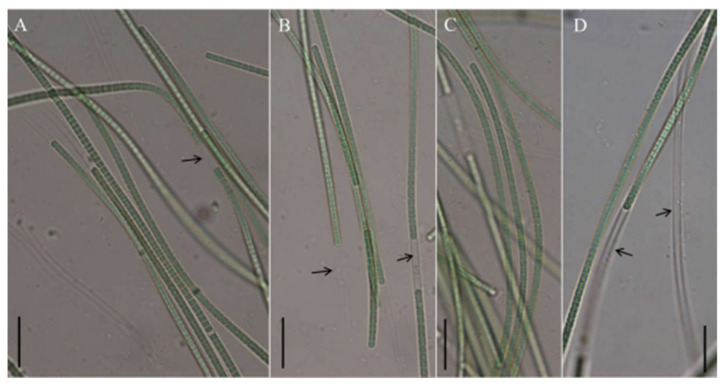
Micrographs of *Neoleptolyngbya chanbaensis* CB-4: (**A**–**D**) filaments. Arrows indicate sheath. Scale bars = 10 μm.

*Neoleptolyngbya chanbaensis* J. X. Chen & F. F. Cai sp. nov. (Figure 4).

Diagnosis: Phylogenetically, *Neoleptolyngbya chanbaensis* forms a well-supported clade with *Neoleptolyngbya gaoqiuensis* (sister species) within the novel genus *Neoleptolyngbya*. It differs from *N. gaoqiuensis* in 16S rRNA gene sequence similarity, 16S–23S ITS secondary structure (D1–D1′ and Box–B helices). The two species, *N. chanbaensis* and *N. gaoqiuensis*, exhibit a sequence dissimilarity of 9.3% in the ITS region (Table 2), exceeding the species delineation threshold of >7%.

Description: Thallus blue-green, or brownish green, free-living in water. Filaments in clusters or solitary, curved, or straight. Sheaths distinct, firm, transparent, colorless, often extending beyond the terminal cells. Trichomes with indistinct cross-wall constrictions and an isopolar morphology. Cell isodiametric, longer or shorter than wide, 1.5–4.0 μm long, 2.1–3.5 μm wide. Parietal thylakoids (7–8 per cell, Figure 3C,D). Granules occasionally present in the cells (Figure 3C,D). Necridia absent. Gas vesicle absent. Apical cells elliptical to obtusely rounded. Reproduction by trichome fragmentation.

Holotype here designated: Dried material of strain CB-4, stored at the Freshwater Algal Herbarium (HBI), Institute of Hydrobiology, Chinese Academy of Sciences (Wuhan, China), under specimen number CB202210.

Type locality: The strain CB-4 was collected from freshwater in Chanba National Wetland Park, Xi’an City, Shanxi Province (34°26′31.61″ N, 108°94′80.24″ E), China.

Habitat: Freshwater.

Reference strain: CB-4 (Institute of Hydrobiology, Chinese Academy of Sciences (Wuhan, China), which houses the Freshwater Algal Herbarium).

Etymology: The name of new species “chanbaensis” refers to the locality where the strain was isolated.

**Figure 5:**
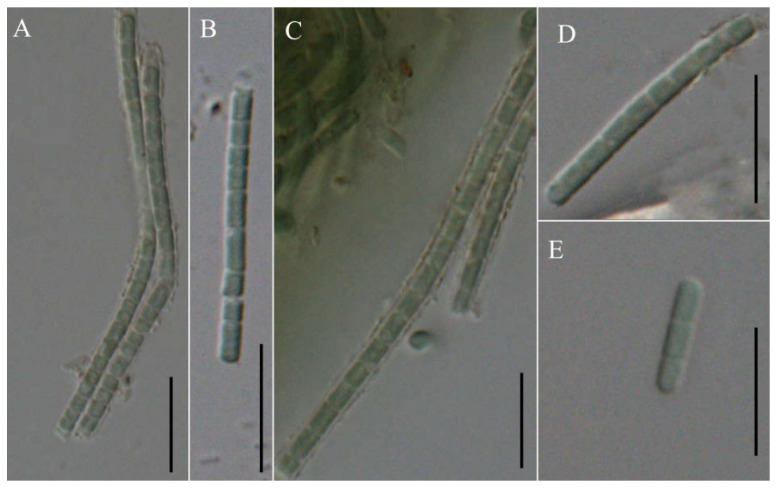
Micrographs of *Pseudoleptolyngbya wuhanensis* LHH-2: (**A**–**D**) single trichome; (**E**) hormogonium. Scale bars = 10 μm.

*Pseudoleptolyngbya wuhanensis* J.X. Chen & F.F. Cai sp. nov. (Figure 5).

Diagnosis: *Pseudoleptolyngbya wuhanensis* was phylogenetically closest to *Pseudoleptolyngbya figueirensis*; however, it differed from this sister species by low 16S rRNA gene sequence similarity and a distinct ITS secondary structure, supporting its recognition as a novel species. In addition, the ITS region of this species shows a notable sequence divergence, exceeding 18% when compared to the other two species (Table 2).

Description: Thallus blue-green. Filaments solitary, straight, unbranched. Sheaths not obvious, mucilaginous diffluent, irregular in outline, colorless, slightly distant to trichomes. Trichomes isopolar, lightly constricted at cross-walls. Cell cylindrical, isodiametric to longer than the width, 1.79–3.40 μm long, 1.84–2.30 μm wide. Apical cells obtuse rounded. Reproduction by trichome fragmentation or hormogonia.

Holotype here designated: Dry material of the strain LHH-2, stored at the HBI, Institute of Hydrobiology, Chinese Academy of Science, Wuhan, China, as specimen No. WH202211.

Type locality: The strain LHH-2 was collected from freshwater in Wuhan City, Hubei province (31°90′60.59″ N, 118°6′60.54″ E), China.

Habitat: Free-floating in freshwater.

Reference strain: LHH-2 (Institute of Hydrobiology, Chinese Academy of Sciences (Wuhan, China), which houses the Freshwater Algal Herbarium).

Etymology: The term “*wuhanensis*” refers to the locality where the strain was isolated.

### 3.3. 16S–23S ITS Region

The 16S–23S ITS sequences of the studied strains were analyzed, and the percent dissimilarity of ITS sequence (including both tRNA genes) between the studied strains and their closely related species was calculated. Three conserved regions (the D1–D1′, Box–B, and the V3 helices) were selected for secondary structure construction.

**Figure 6:**
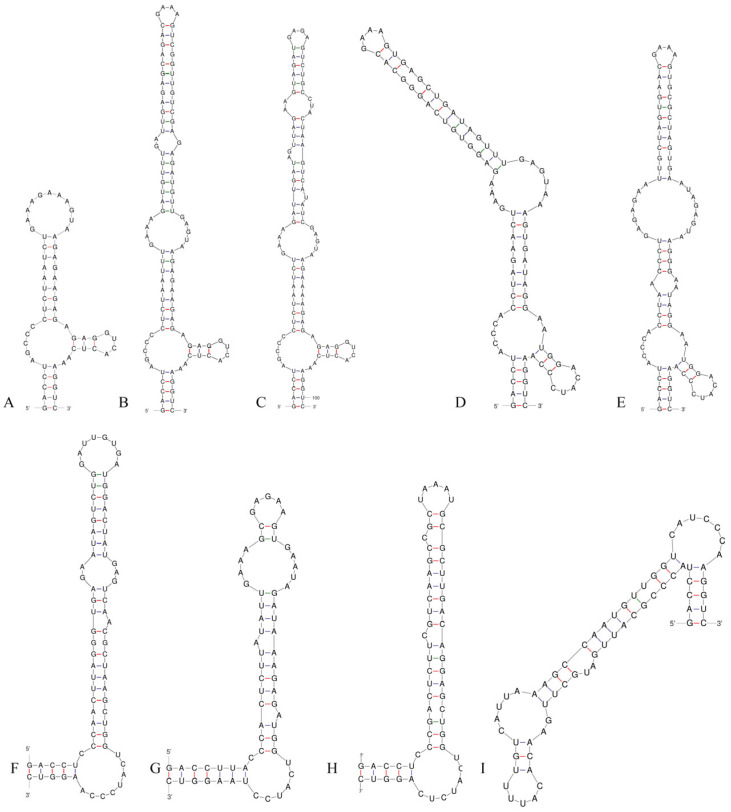
D1–D1′ helices of the 16S–23S ITS for the studied strains and comparison taxa: (**A**) *Pseudoleptolyngbya wuhanensis* LHH-2; (**B**) *Pseudoleptolyngbya figueirensis* LEGE 16533; (**C**) *Pseudoleptolyngbya monchiquensis* LEGE 16651; (**D**) *Neoleptolyngbya chanbaensis* CB-4; (**E**) *Neoleptolyngbya gaoqiuensis* GQSK-2; (**F**) *Leptolyngbyopsis coimbrensis* LEGE 16524; (**G**) *Limnolyngbya circumcreta* CHAB4449; (**H**) *Chroakolemma opacum* strain 701; (**I**) *Stenomitos rutilans* HA7619-LM2.

**Figure 7:**
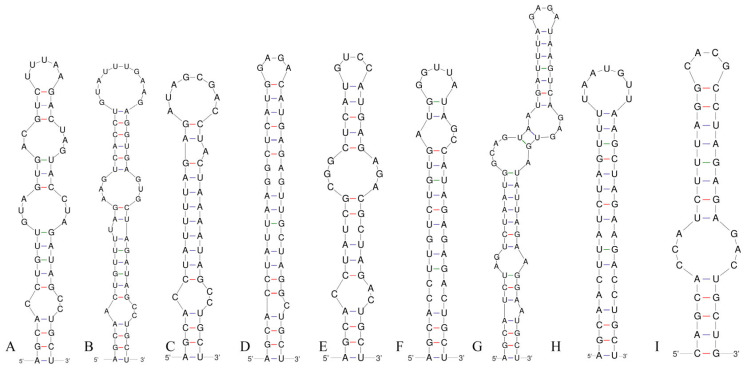
Box–B helices of the 16S–23S ITS for the studied strains and comparison taxa: (**A**) *Pseudoleptolyngbya wuhanensis* LHH-2; (**B**) *Pseudoleptolyngbya figueirensis* LEGE 16533; (**C**) *Pseudoleptolyngbya monchiquensis* LEGE 16651; (**D**) *Neoleptolyngbya chanbaensis* CB-4; (**E**) *Neoleptolyngbya gaoqiuensis* GQSK-2; (**F**) *Leptolyngbyopsis coimbrensis* LEGE 16524; (**G**) *Limnolyngbya circumcreta* CHAB4449; (**H**) *Chroakolemma opacum* strain 701; (**I**) *Stenomitos rutilans* HA7619-LM2.

**Figure 8:**
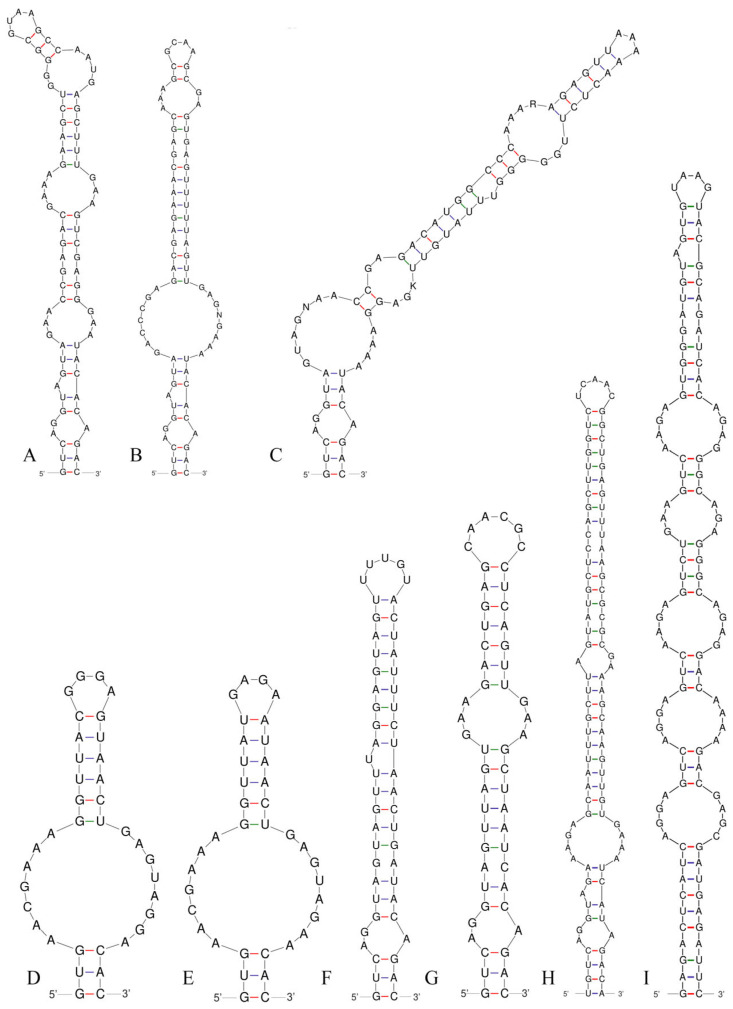
V3 helices of the 16S–23S ITS for the studied strains and comparison taxa: (**A**) *Pseudoleptolyngbya wuhanensis* LHH-2; (**B**) *Pseudoleptolyngbya figueirensis* LEGE 16533; (**C**) *Pseudoleptolyngbya monchiquensis* LEGE 16651; (**D**) *Neoleptolyngbya chanbaensis* CB-4; (**E**) *Neoleptolyngbya gaoqiuensis* GQSK-2; (**F**) *Leptolyngbyopsis coimbrensis* LEGE 16524; (**G**) *Chroakolemma opacum* strain 701; (**H**) *Stenomitos rutilans* HA7619-LM2; (**I**) *Limnolyngbya circumcreta* CHAB4449.

Analysis of the secondary structure of the D1–D1′ helix revealed unique structural features in *Neoleptolyngbya* (Figure 6). The D1–D1′ helix of *Neoleptolyngbya* is unique among these genera, with a stem (5′UGGACAUCCCA′3) originating from the first lateral bulge (5:2) and opposing a sequence of five free residues (5′ACCCA′3) (Figure 6D,E). Beyond the first lateral bulge, the D1–D1′ helix structures of the two *Neoleptolyngbya* species exhibit entirely different configurations. In *Neoleptolyngbya chanbaensis* CB-4, the first lateral bulge leads through a 9 bp stem and a 4:6 bp bilateral bulge, terminating in a 15 bp stem region (Figure 6D). In contrast, *Neoleptolyngbya gaoqiuensis* GQSK-2 features a 1:2 bp bilateral bulge and a 7:8 bp bilateral bulge after the first lateral bulge, followed by a 12 bp stem region (Figure 6E). The closely related genus *Leptolyngbyopsis* has a 1:8 bp bilateral bulge, followed by an unpaired 3′ nucleotide, and a 2:3 bp bilateral bulge, which is closed by an 8 bp terminal loop (5′GAUUGUGA′3) (Figure 6F). The Box–B helix in *Neoleptolyngbya gaoqiuensis* GQSK-2 comprises a 4 bp (AGCA) basal stem, a 1:2 nucleotide asymmetry, a 3:3 bilateral bulge, and ends in a 4 bp terminal loop (GUCC) (Figure 7E). The Box–B helix of *Neoleptolyngbya chanbaensis* CB-4 also consists of a 4 bp (AGCA) helix in the base of the stem, followed by an unpaired single nucleotide on the 3′ side, and ends in a 4 bp terminal loop (GAGA) (Figure 7D). In contrast, the Box–B structure of the closely related genus *Leptolyngbyopsis* exhibits variations in both length and structural features. Specifically, it is characterized by an extended 15 bp basal stem, which extends into a 1:2 bp bilateral bulge and culminates in a 5 bp terminal loop (GGUUA) (Figure 7F). The V3 helix comparison between *Neoleptolyngbya* and closely related genus *Leptolyngbyopsis* revealed significant structural variations (Figure 8). The V3 helix in *Neoleptolyngbya* comprises a 3 bp basal stem, a 7:8 bp bilateral bulge, and ends in a 4 bp terminal loop (Figure 8D,E). In contrast, the V3 structure of *Leptolyngbyopsis* showed a 3 bp basal stem, a 2:1 bp bilateral bulge, an unpaired single nucleotide on the 5′ side, and a 5 bp terminal loop (Figure 8F). The V3 helix structures of the two *Neoleptolyngbya* species exhibit a similar pattern, differing only in their nucleotide sequences. The percent dissimilarity of ITS between *Neoleptolyngbya gaoqiuensis* GQSK-2 and *Neoleptolyngbya chanbaensis* CB-4 is 9.3% (Table 2).

The analysis of the ITS secondary structures of *Pseudoleptolyngbya wuhanensis* LHH-2 in comparison to the other two *Pseudoleptolyngbya* species revealed unique features for *Pseudoleptolyngbya wuhanensis* LHH-2 (Figure 6A–C). The D1–D1′ helix of three *Pseudoleptolyngbya* species has a stem (5′GAGGUCACUC3′) originating from the first lateral bulge (5:3) and opposing a sequence of five free residues (5′AGCCC3′), and followed by a large terminal loop containing 11 bp bases in *Pseudoleptolyngbya wuhanensis* LHH-2 (but two bilateral bulges in *Pseudoleptolyngbya figueirensis* LEGE 16533, and two bilateral bulges and two unpaired single nucleotides in *Pseudoleptolyngbya monchiquensis* LEGE 16651). The Box–B helices in *Pseudoleptolyngbya* were conserved at the basal stem regions while demonstrating divergence in both sequences and structural features. The Box–B helix of *Pseudoleptolyngbya wuhanensis* LHH-2 has three bilateral bulges of 1:2, 3:3 and 2:3 nt, and the terminal loop contains 5 bp bases (UUUAA) (Figure 7A). The Box–B helix of *Pseudoleptolyngbya figueirensis* LEGE 16533 has a 1:2 bilateral bulge, an unpaired single nucleotide on the 5′ side, a 3:3 bilateral bulge, and a large terminal loop containing 10 bp bases (Figure 7B). The Box–B helix of *Pseudoleptolyngbya monchiquensis* LEGE 16651 consists a 1:2 bilateral bulge, an unpaired 3′ nucleotide, and an 8 bp terminal loop (Figure 6C). The V3 helices of *Pseudoleptolyngbya* varied in sequence, structure, and length. The V3 helix of *Pseudoleptolyngbya wuhanensis* LHH-2 was completely different from that of the other two *Pseudoleptolyngbya* species (Figure 8A–C). The base stem of *Pseudoleptolyngbya wuhanensis* LHH-2 was made up of a 3 bp helix, a 2:1 bp bilateral bulge, an unpaired single nucleotide on the 5′ side, three bilateral bulges of 3:3, 4:3 and 2:4 nt, and a 4 bp terminal loop (GUAA) (Figure 8A). The V3 helix of *Pseudoleptolyngbya figueirensis* LEGE 16533 has a 2:1 bilateral bulge, an unpaired 5′ nucleotide, a 7:8 bilateral bulge, a 3:2 bilateral bulge, and a 4 bp terminal loop (GCAA) (Figure 8B). The V3 helix of *Pseudoleptolyngbya monchiquensis* LEGE 16651 consists of four bilateral bulges of 2:1, 7:4, 2:3, and 4:3 nt, an unpaired single nucleotide on the 3′ side, and a terminal loop containing 3 bp bases (AAA) (Figure 8C). The percent dissimilarity of ITS of *Pseudoleptolyngbya wuhanensis* LHH-2 to the other two *Pseudoleptolyngbya* species is in the range of 18.8–21.8% (Table 2).

## 4. Discussion

Morphological characteristics alone are insufficient to delineate genus and species boundaries in certain cyanobacterial taxa, particularly the thin filamentous cyanobacteria. Thus, molecular approaches have become the primary method for cyanobacterial identification [17,36]. In modern classification systems, the description of a novel cyanobacterial genus requires evidence of a well-supported monophyletic phylogenetic position, along with clear evolutionary discontinuity from the nearest sister clade [15,17,26,37,38,39].

Using a polyphasic taxonomic approach, we describe strains GQSK-2 and CB-4 as two new species, *Neoleptolyngbya gaoqiuensis* and *Neoleptolyngbya chanbaensis*, within the novel genus *Neoleptolyngbya* (family Leptolyngbyaceae). Phylogenetically, *Neoleptolyngbya* is closely related to *Leptolyngbyopsis* (with strong support; Figure 1) within Leptolyngbyaceae. *Leptolyngbyopsis* is a cryptic genus with diverse filament morphologies; *Neoleptolyngbya* can be morphologically distinguished from *Leptolyngbyopsis* only by its wider cell width: *Leptolyngbyopsis* has a cell width of 1.7–2 μm, while *Neoleptolyngbya* has a cell width of 2.0–3.6 μm (Table 3). Additionally, *Neoleptolyngbya* can also be distinguished from *Leptolyngbyopsis* and other closely related genera by its high 16S rRNA p-distance (above the 5.8% threshold), which falls below the 95% genus delineation threshold proposed by Yarza et al. [40]. These results further support the establishment of *Neoleptolyngbya* as a new genus. Variations in the length of conserved domains in the 16S–23S rDNA ITS region and differences in ITS secondary structures (e.g., D1–D1′, Box–B, and V3 helices) have been used as autapomorphies to characterize and support novel cryptic genera or species [17,26,38,39,41,42,43,44]. Comparative analyses of predicted ITS secondary structures further confirms genus-level divergence among phylogenetically related taxa. The D1–D1′ helix, which was considered to be highly conservative, was significantly different between *Neoleptolyngbya* and related taxa. The two A bases and the stem-loop region (UGGACAUCCCA) in opposition to the first lateral bulge can serve as a diagnostic feature distinguishing *Neoleptolyngbya* from other genera within the family. Additionally, the large 7:8 bp bilateral bulge in the V3 helix of *Neoleptolyngbya* is a unique feature not observed in other closely related genera of Leptolyngbyaceae.

The 16S rRNA gene sequence similarity among *Neoleptolyngbya* species does not meet the 98.7% species delineation threshold defined by Yarza et al. [40], providing molecular evidence for their taxonomic distinction. Moreover, the D1–D1′ and Box–B helices of *Neoleptolyngbya gaoqiuensis* exhibit pronounced differences from those of *N. chanbaensis*. The V3 helix structures of the two *Neoleptolyngbya* species display similar configurations while differing in their nucleotide sequences. Furthermore, the percent dissimilarity in the 16S–23S ITS region is valuable for cyanobacterial species delimitation. The 16S–23S ITS region shows a 9.3% dissimilarity between *Neoleptolyngbya gaoqiuensis* GQSK-2 and *N. chanbaensis* CB-4, exceeding >7% threshold for species separation [17,21,41,45,46].

*Pseudoleptolyngbya* is a newly established genus presently known to contain two species, namely, *Pseudoleptolyngbya figueirensis* and *Pseudoleptolyngbya monchiquensis*, both isolated from artificial freshwater fountains in Portugal [26]. The new *Pseudoleptolyngbya* species, *Pseudoleptolyngbya wuhanensis*, here described was also found living in a freshwater habitat similar to the previously described species. From a genetic perspective, *Pseudoleptolyngbya wuhanensis* is clearly distinct from its sister species: it exhibits a >4.4% difference in 16S rRNA gene sequence and a >18% difference in the 16S–23S rRNA ITS region compared to the other *Pseudoleptolyngbya* species. Moreover, when the new species in this study is compared with its closely related species *Pseudoleptolyngbya monchiquensis*, the difference in 16S rRNA is greater than 5.3%, and the difference in ITS is greater than 21%.

The ITS secondary structure exhibits distinct structural differences among the three species. *Pseudoleptolyngbya wuhanensis* shares a similar basal lateral bulge in the D1–D1′ helix with the other two *Pseudoleptolyngbya* species. However, the structures posterior to the base are entirely different, manifested in the following details: *Pseudoleptolyngbya wuhanensis* has no bilateral bulge in the central region and only has a 11 bp terminal loop, whereas *Pseudoleptolyngbya figueirensis* possesses two bilateral bulges and a 4 bp terminal loop, and *Pseudoleptolyngbya monchiquensis* exhibits two bilateral bulges, one unpaired single nucleotide, one 2 bp unilateral bulge, and a 4 bp terminal loop. Additionally, the Box–B helix of the three species exhibit differences, apart from the shared 1:2 bp bilateral bulge at the base, *Pseudoleptolyngbya wuhanensis* possesses two additional bilateral bulges and a 5 bp terminal loop. *Pseudoleptolyngbya figueirensis* has one unilateral bulge, an unpaired single nucleotide on the 5′ side, and a 10 bp terminal loop, while *Pseudoleptolyngbya monchiquensis* contains one unpaired single nucleotide on the 3′ side and a 8 bp terminal loop. The V3 helix structures of the three species are completely distinct, with each species representing a distinct type. Despite morphological similarities between *Pseudoleptolyngbya wuhanensis* and the other *Pseudoleptolyngbya* species, genetic analyses of the 16S rRNA and ITS regions confirm its status as a novel taxon.

The descriptions of all novel taxa were conducted using a polyphasic approach, encompassing morphological characterization and comparisons with phylogenetically related taxa, genetic analyses based on 16S rRNA and 16S–23S rRNA, 16S rRNA phylogenetic analysis, and ecological characteristics. In summary, the strains GQSK-2 and CB-4 were described as two new species, *Neoleptolyngbya gaoqiuensis* sp. nov. and *Neoleptolyngbya chanbaensis* sp. nov., in a new genus, *Neoleptolyngbya* gen. nov.; the strain LHH-2 was described as one new species, *Pseudoleptolyngbya wuhanensis* sp. nov. Significant gaps remain in our understanding of global cyanobacterial diversity, with numerous species awaiting discovery and formal description. The taxonomy of cyanobacteria will undoubtedly be refined in the future. China, with its vast territory, is one of the world’s most biodiverse countries, and a large number of cyanobacterial resources remain unexplored. Given the substantial proportion of undiscovered cyanobacterial diversity, we will continue to conduct systematic surveys across various regions to identify novel taxa, thereby enriching our knowledge of cyanobacterial biodiversity.

## Figures and Tables

**Table 1:** Sequence similarity comparison of the 16S rRNA gene between the studied strains and closely related taxa. Similarity = [1 − (*p*-distance)] × 100.

Strains	1	2	3	4	5	6	7	8	9	10
**1. *Neoleptolyngbya chanbaensis* CB-4 clone1**	ID									
**2. *Neoleptolyngbya chanbaensis* CB-4 clone2**	1	ID								
**3. *Neoleptolyngbya gaoqiuensis* GQSK-2 clone1**	0.982	0.982	ID							
**4. *Neoleptolyngbya gaoqiuensis* GQSK-2 clone2**	0.982	0.982	1	ID						
**5. *Pseudoleptolyngbya wuhanensis* LHH-2 clone1**	0.895	0.895	0.894	0.894	ID					
**6. *Pseudoleptolyngbya wuhanensis* LHH-2 clone2**	0.895	0.895	0.894	0.894	1	ID				
7. *Chroakolemma opacum* strain 701 clone 3	0.914	0.914	0.909	0.909	0.884	0.884	ID			
8. *Leptolyngbyopsis coimbrensis* LEGE 16524	0.942	0.942	0.935	0.935	0.891	0.891	0.923	ID		
9. *Limnolyngbya circumcreta* CHAB4449 clone 2	0.92	0.92	0.917	0.917	0.875	0.875	0.923	0.912	ID	
10. *Pseudoleptolyngbya monchiquensis* LEGE 16651	0.907	0.907	0.906	0.906	0.947	0.947	0.896	0.9	0.888	ID
11. *Pseudoleptolyngbya figueirensis* LEGE 16533	0.923	0.923	0.918	0.918	0.957	0.957	0.904	0.911	0.903	0.971

**Table 2:** Percent dissimilarity based on 16S–23S ITS sequence among the studied strains and the closest sister taxa.

Strains	1	2	3	4	5
1. ***Neoleptolyngbya chanbaensis*** **CB-4**	0				
2. ***Neoleptolyngbya gaoqiuensis*** **GQSK-2**	9.3				
3. *Leptolyngbyopsis coimbrensis* LEGE 16524	29.7	31.9			
4. ***Pseudoleptolyngbya wuhanensis*** **LHH-2**	37.9	38.7	38.9		
5. *Pseudoleptolyngbya monchiquensis* LEGE 16651	39.5	40.8	37.8	21.8	
6. *Pseudoleptolyngbya figueirensis* LEGE 16533	41.6	41.8	41.6	18.8	23.9

**Table 3:** Comparison of the characteristics of the studied species with closely related species.

Characteristics	*Neoleptolyngbya gaoqiuensis*	*Neoleptolyngbya chanbaensis*	*Leptolyngbyopsis coimbrensis*	*Pseudoleptolyngbya wuhanensis*	*Pseudoleptolyngbya figueirensis*	*Pseudoleptolyngbya monchiquensis*
Cell length (μm)	1.3–4.1	1.5–4.0	1.6–2.5	1.79–3.40	2.3–3.3	2.4–3.4
Cell width (μm)	2.0–3.6	2.1–3.5	1.7–2	1.84–2.30	1.6–2	1.6–1.9
Cell content	Homogeneous or granules	Homogeneous or granules	Homogeneous	Homogeneous	Homogeneous	Homogeneous
Thallus	Bright blue-green	Blue-green, or brownish green	Pale green	Blue-green	Olive-green	Olive-green to brownish
Filaments	Solitary or in clusters, straight, or curved	Solitary or in clusters, straight, or curved	Short or long, solitary or forming entangled clusters, flexuous	Solitary, straight, or slender, unbranched	Solitary or densely entangled	Solitary or entangled
Sheath	Firm, transparent, colorless	Firm, transparent, colorless	Very thin, firm, joined to the trichome, colorless	Mucilaginous diffluent	Very thin, joined to the trichome, firm, colorless	Mucilaginous diffluent, very thin
Habitat	Freshwater	Freshwater	Planktonic in fresh water	Freshwater	Planktonic in fresh water	Planktonic in fresh water
Geography	China	China	Portugal	China	Portugal	Portugal

## Data Availability

The raw data supporting the conclusions of this article will be made available by the authors on request.

## Supplementary Materials

The following supporting information can be downloaded at: https://www.mdpi.com/article/10.3390/microorganisms14010072/s1, Table S1: 16S rRNA gene for phylogeny.
